# TDP-43 mislocalization drives neurofilament changes in a novel model of TDP-43 proteinopathy

**DOI:** 10.1242/dmm.047548

**Published:** 2021-02-11

**Authors:** Rachel Atkinson, Jacqueline Leung, James Bender, Matthew Kirkcaldie, James Vickers, Anna King

**Affiliations:** Wicking Dementia Research and Education Centre, University of Tasmania, Medical Science Precinct, 17 Liverpool Street, Hobart, Tasmania 7000, Australia

**Keywords:** TDP-43, Amyotrophic lateral sclerosis, Frontotemporal lobar degeneration, Neurodegeneration, Disease model, Visual system

## Abstract

Mislocalization of the TAR DNA-binding protein 43 (TDP-43; encoded by *TARDBP*) from the nucleus to the cytoplasm is a common feature of neurodegenerative conditions such as amyotrophic lateral sclerosis (ALS) and frontotemporal lobar degeneration (FTLD). The downstream *in vivo* cellular effects of this mislocalization are not well understood. To investigate the impact of mislocalized TDP-43 on neuronal cell bodies, axons and axonal terminals, we utilized the mouse visual system to create a new model of TDP-43 proteinopathy. Mouse (C57BL/6J) retinal ganglion cells (RGCs) were transduced with GFP-tagged human wild-type TDP-43 (hTDP-WT-GFP) and human TDP-43 with a mutation in the nuclear localization sequence (hTDP-ΔNLS-GFP), to cause TDP-43 mislocalization, with ∼60% transduction efficiency achieved. Expression of both hTDP-WT-GFP and hTDP-ΔNLS-GFP resulted in changes to neurofilament expression, with cytoplasmic TDP-43 being associated with significantly (*P*<0.05) increased neurofilament heavy expression in the cell soma, and both forms of altered TDP-43 leading to significantly (*P*<0.05) decreased numbers of neurofilament-positive axons within the optic nerve. Alterations to neurofilament proteins were associated with significantly (*P*<0.05) increased microglial density in the optic nerve and retina. Furthermore, expression of hTDP-WT-GFP was associated with a significant (*P*<0.05) increase in pre-synaptic input into RGCs in the retina. The current study has developed a new model that allows detailed examination of alterations to TDP-43 and will contribute to the knowledge of TDP-43-mediated neuronal alterations and degeneration.

## Abstract (precis)

**Summary:** We examined the cellular effects of mislocalization of TDP-43, a key pathological protein in amyotrophic lateral sclerosis and frontotemporal dementia, using the eye as a model and demonstrated axonal cytoskeleton alterations.

## INTRODUCTION

Amyotrophic lateral sclerosis (ALS) and frontotemporal lobar degeneration (FTLD) are characterized by the degeneration of distinct nerve cell populations in specific areas of the brain and spinal cord. A common pathological feature of these diseases is TAR DNA-binding protein 43 (TDP-43; encoded by *TARDBP*) mislocalization from the nucleus to the cytoplasm of neurons (Neumann et al., 2006), which occurs in 97% of ALS cases and in 45% of FTLD cases (Ling et al., 2013), and includes both sporadic forms and individuals with mutations in *C9orf72* and progranulin genes (DeJesus-Hernandez et al., 2011; Cairns et al., 2007). Although studies in primary neuronal culture suggest that mislocalization of TDP-43 is an important driver of cellular toxicity (Barmada et al., 2010), the exact relationship between TDP-43 mislocalization and cellular pathology is unclear.

In healthy cells, TDP-43 is predominantly a nuclear protein with important roles in regulation of RNA. It binds to RNA through its two RNA recognition motifs (RRM1 and RRM2), and is able to shuttle between the nucleus and cytoplasm due to its nuclear export sequence (NES) and nuclear localization sequence (NLS) (as reviewed in Lee et al., 2012). The protein has distinct nuclear and cytoplasmic functions. Within the nucleus, TDP-43 plays a variety of roles in mRNA splicing and processing of microRNAs. Within the cytoplasm, TDP-43 localizes to stress granules, where it is thought to be involved in trafficking and stabilizing mRNA (Colombrita et al., 2015; Dewey et al., 2011; McDonald et al., 2011). TDP-43 has also been demonstrated to play a role in distal axons and dendrites, where it localizes to RNA granules, and has been proposed to be involved in RNA trafficking throughout these neurites (Alami et al., 2014).

Histological examination of TDP-43 in human ALS and FTLD tissue demonstrates a variety of inclusions, including nuclear and cytoplasmic aggregates that are phosphorylated and ubiquitinated (Mackenzie and Neumann, 2016). Mislocalization of TDP-43, where there is marked depletion from the nucleus and accumulation in the cytoplasm, is the most common finding in TDP-43-related disease and is proposed to result in both a loss of function of nuclear TDP-43 and a toxic gain of function, where cytoplasmic TDP-43 aberrantly sequesters other RNA binding proteins (Lee et al., 2012). Phosphorylated TDP-43 also accumulates in axons and dendrites of somatomotor neurons as skein-like and dash-like aggregates (Braak et al., 2010; Brettschneider et al., 2014). These alterations to TDP-43 are associated with a number of cellular changes, including dystrophic neurites in cortex and hippocampus in FTLD with TDP-43 inclusions (Hatanpaa et al., 2008), and severe loss of axons and white matter in both FTLD and ALS (Armstrong, 2017; Fatima et al., 2015). Neurofilament alterations are also a common feature of ALS; for example, neurofilament proteins become aggregated into spheroids within proximal motor neuron axons in ALS cases (Corbo and Hays, 1992; Hirano et al., 1984a,b). The link between these alterations and TDP-43 pathology have not yet been well researched. It is known that TDP-43 and other pathogenic proteins associated with ALS play roles in regulating the mRNA for neurofilament light (*Nefl*; encoding NFL) (Strong et al., 2007). We have previously shown that, in an NFL knockout mouse model, TDP-43 was abnormally increased, without abnormal localization or aggregate formation (Liu et al., 2015).

There have been a number of studies in which TDP-43 expression has been altered through disease-related mutations (for example, Arnold et al., 2013) or by manipulating the TDP-43 NLS to cause mislocalization (for example, Han et al., 2013; Barmada et al., 2010; Walker et al., 2015; Igaz et al., 2011). *In vitro* studies demonstrate that alterations to TDP-43 expression and localization lead to altered neuron morphology (Han et al., 2013) and cellular toxicity (Tripathi et al., 2014; Barmada et al., 2010), while *in vivo* studies show axonal dieback (Walker et al., 2015), corticospinal tract degeneration and neuronal loss independent of cytoplasmic inclusions (Igaz et al., 2011), and motor axon degeneration without cytoplasmic mislocalization (Arnold et al., 2013). However, limitations of these studies are that they either allow examination of structural changes (*in vitro*) or changes in the complex environment of the nervous system (*in vivo*) but not both.

**Fig. 1.**
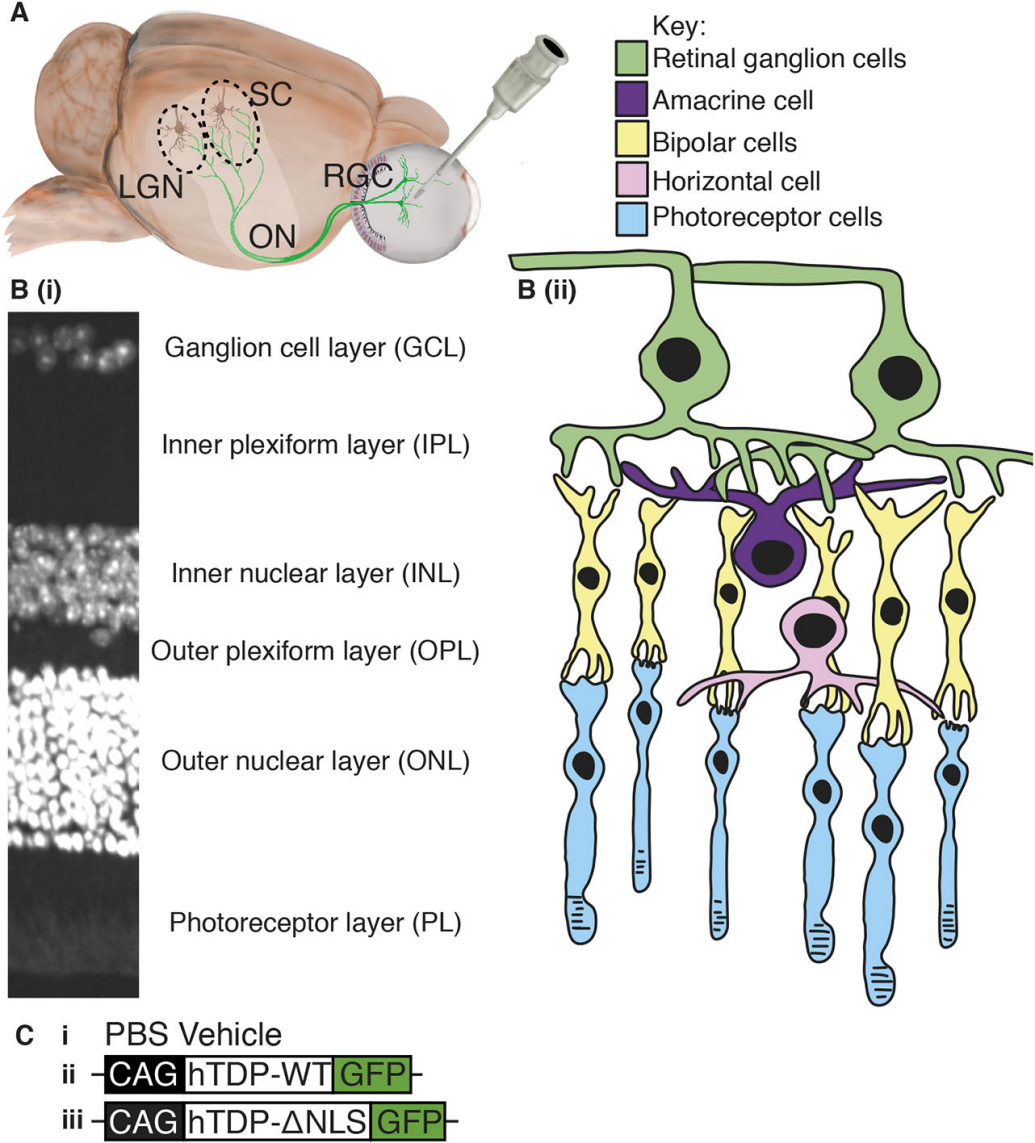
**Eye model and retina layers.** (A) Schematic of the eye model, showing injection into the vitreous humour. Green neurons indicate retinal ganglion cells (RGC), passing through the optic nerve (ON) to both the superior colliculus (SC) and the lateral geniculate nucleus (LGN). (B) DAPI-stained retinal cross section (i) and drawing (ii), showing layers and cell types within the retina. (C) Schematic indicating the constructs injected into the eye: PBS (vehicle) (i), AAV2 constructs containing human wild-type TDP-43 (hTDP-WT; ii) and human TDP-43 with mutated nuclear localization sequence (hTDP-ΔNLS; iii), both under the CAG promoter and tagged with green fluorescent protein (GFP) at the C-terminus.

In order to overcome these limitations and to further understand the cellular consequences of TDP-43 mislocalization to the cytoplasm in individual neurons, we have developed a novel *in vivo* central nervous system (CNS) model using the visual system of mice. The visual system has been utilized to examine ultrastructural changes in axons following injury (Knoferle et al., 2010) as it offers unique access to the CNS with relative ease. Adeno-associated viruses (AAVs) can be used to efficiently transduce retinal ganglion cells (RGCs) with foreign genes (Grant et al., 1997) via intraocular injection (Fig. 1A). The RGCs are a useful population of neurons for modelling disease because they are well-characterized and their unique location in the retina (Fig. 1B) allows easy access for transduction of their soma, as well as analysis of their synaptic inputs, dendrites, axons in the optic nerve and terminals in the lateral geniculate nucleus (LGN) and superior colliculus (SC) (Fig. 1A). Although RGCs are not specifically implicated in ALS, our model relies on the hypothesis that it is the development of pathology that is cell-type specific and that the downstream effects of pathology are universal.

In the current study, TDP-43 with a defective NLS was expressed in RGCs to create a new TDP-43 disease model. RGCs of wild-type (WT) C57BL/6J mice were transduced with AAV2 constructs containing the human TDP-43 sequence with a defective NLS, fused to GFP (hTDP-ΔNLS-GFP), and human WT TDP-43, fused to GFP (hTDP-WT-GFP) (Fig. 1C). The downstream effects of altered TDP-43 on the cellular features of RGCs, including expression of neurofilament proteins, was examined in these CNS neurons.

## RESULTS

### Establishment of viral transduction of RGCs using intraocular injection

A pilot study using serial dilutions of hTDP-WT-GFP and hTDP-ΔNLS-GFP AAV2 injected into the mouse retina demonstrated that 1 µl of virus at 3.375×10^12^ genome copies (GC)/ml was the optimal concentration to ensure transduction efficiency above 40% (Fig. S1).

**Fig. 2.**
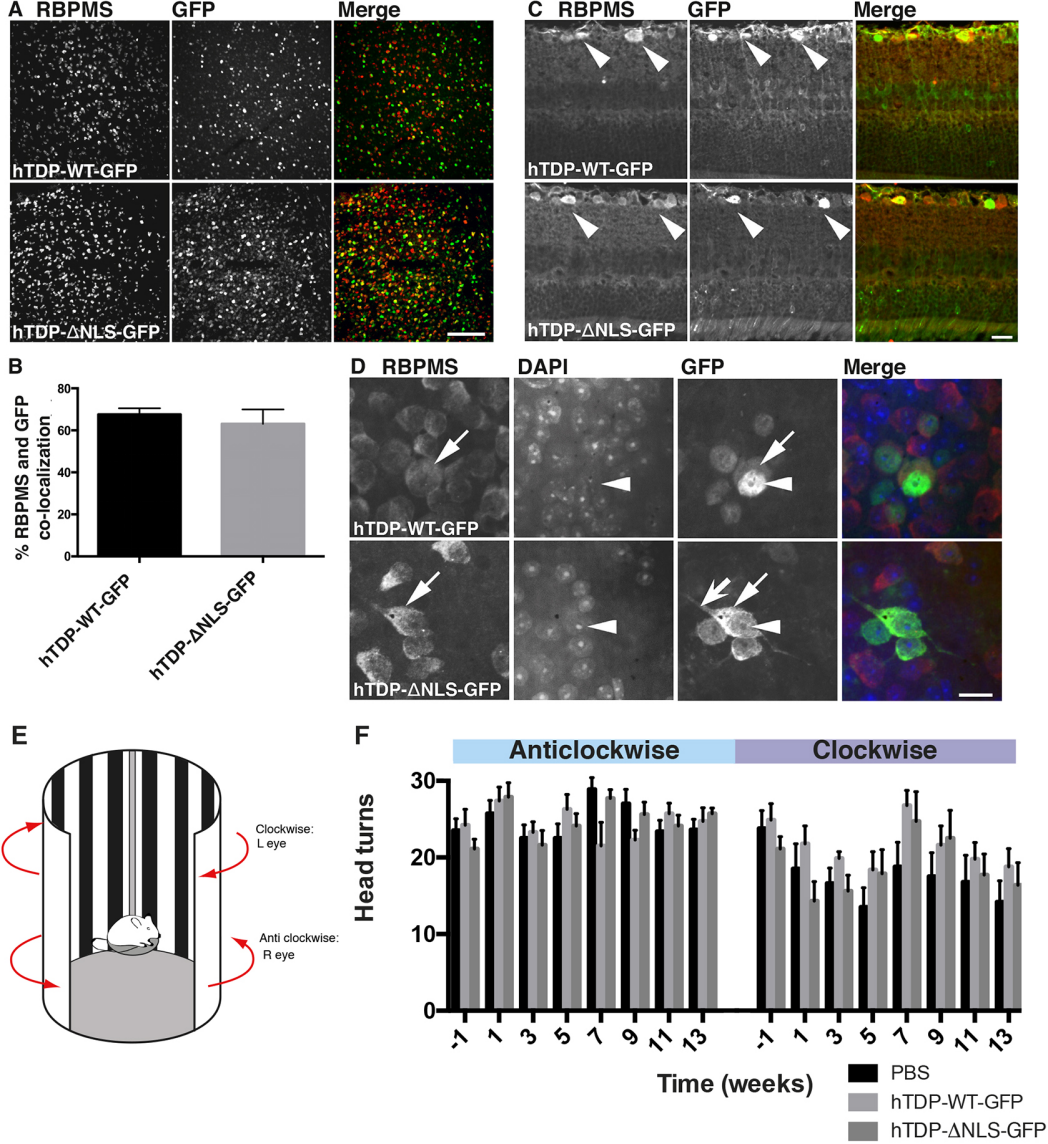
**Characterization of TDP-43 construct expression in the retina.** (A) Wholemount retina from mice injected with hTDP-WT-GFP and hTDP-ΔNLS-GFP immunolabelled for RBPMS (red) and GFP (green). (B) The percentage of colocalizing GFP and RBPMS RGC bodies was quantified to provide an estimate of viral transduction efficiency. Results are mean±s.e.m., *n*=5 per treatment group. (C) Cross-sectioned retinas from mice injected with hTDP-WT-GFP and hTDP-ΔNLS-GFP immunolabelled for RBPMS (red) and GFP (green), showing GFP-positive RGC bodies in the upper ganglion cell layer (arrowheads), but no GFP in the other cellular layers of the retina. (D) Wholemount retina from mice injected with hTDP-WT-GFP and hTDP-ΔNLS-GFP immunolabelled for RBPMS (red) and GFP (green) and stained with DAPI (blue). Top row shows hTDP-WT-GFP in nuclei (arrowheads) and at low levels in the cytoplasm (arrows). Bottom row shows hTDP-ΔNLS-GFP in cell bodies (arrows), but at lower levels in the nuclei (arrowhead), and in proximal neurites (barbed arrow). (E) Schematic of the rig used to test optomotor response. (F) Quantitation of the number of head turns in anticlockwise (untreated) and clockwise (treated) direction for each treatment group eyes over time (weeks). Values are mean±s.e.m.; *n*=7 per treatment group. Data were analysed with linear mixed effects models. Scale bars: 100 µm (A), 20 µm (C,D).

hTDP-WT-GFP, hTDP-ΔNLS-GFP AAV2 or PBS vehicle were injected into the left eye of C57BL/6J mice, and the effects analysed after 3 months. Viral transduction efficiency of RGCs in the retina was confirmed in wholemount retina, with GFP and RBPMS (a selective marker of RGCs; Rodriguez et al., 2014) colocalization (Fig. 2A) demonstrating that 67.6±2.9% of RGCs were transduced in hTDP-WT-GFP retinas and 63.1±6.9% of RGCs were transduced in hTDP-ΔNLS-GFP retinas (Fig. 2B).

GFP fluorescence was used to locate hTDP-WT-GFP and hTDP-ΔNLS-GFP within RGCs. Cross-sectioned retinas showed GFP labelling restricted to the RGC layer for both hTDP-WT-GFP and hTDP-ΔNLS-GFP (Fig. 2C), with no GFP in any other layers. Wholemount and cross-sectioned retinas showed GFP fluorescence in RBPMS-positive RGCs (Fig. 2C,D). In hTDP-WT-GFP retinas, fluorescence was localized to RGC nuclei, with low expression in the cytoplasm. In hTDP-ΔNLS-GFP retinas, fluorescing RGCs had high expression throughout the soma (Fig. 2D). In ∼12% of RGCs expressing hTDP-ΔNLS-GFP, fluorescence extended to proximal neurites. TDP-43 aggregation induced by overexpressing hTDP-ΔNLS-GFP and hTDP-WT-GFP was determined by examining the distribution of GFP fluorescence within cells (Fig. 2D) and phosphorylated TDP-43 (Fig. S2); however, there was no evidence of aggregates in RGCs.

### Visual acuity is not altered by treatment

Functional changes to visual acuity following intravitreal injections were determined by the optomotor response (Fig. 2E), comparing whether treatment to the left eye (hTDP-WT-GFP, hTDP-ΔNLS-GFP or vehicle; assessed by clockwise head turning) altered the number of head turns over time compared to untreated eyes (assessed by anticlockwise head turning). These results demonstrated that there was no evidence of an interaction between treatment and other factor variables (time and head turns in anticlockwise or clockwise directions; *P*>0.05) (Fig. 2F). The number of clockwise head turns (treated eye) decreased for all treatments (*P*<0.05), potentially indicating an effect of the injection itself.

### Altered TDP-43 induces an inflammatory response

**Fig. 3.**
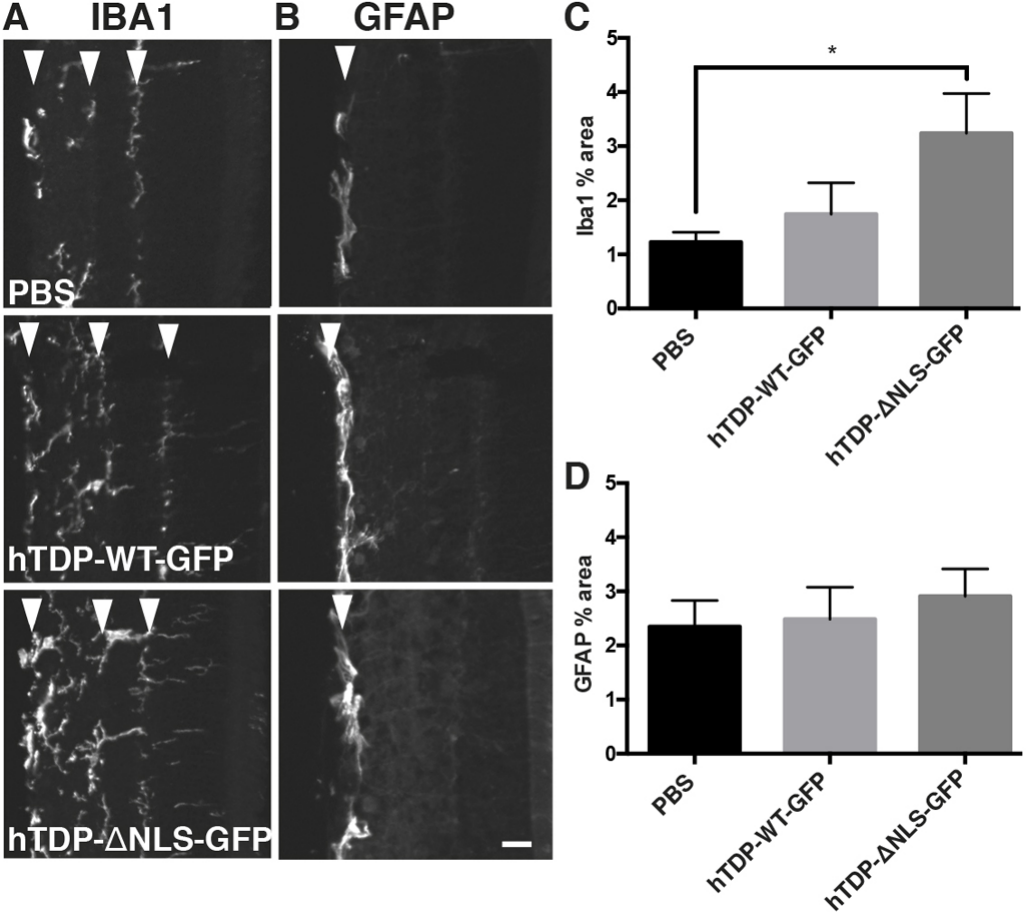
**Microglial alterations associated with transduction with altered TDP-43.** (A,B) Cross-sectioned retinas from mice injected with vehicle (PBS), hTDP-WT-GFP or hTDP-ΔNLS-GFP immunolabelled with Iba1 (A) and GFAP (B). Arrowheads indicate Iba1 immunolabelling in the ganglion cell layer (GCL), inner nuclear layer (INL) and outer nuclear layer (ONL) (left to right in A) and GFAP immunolabelling in the GCL only (B). (C,D) The percentage area occupied by segmented Iba1 (C) and GFAP (D) staining was quantified. Values are mean±s.e.m., *n*=5 per treatment group. *P*-values are from one-way ANOVAs with Tukey post hoc tests; *P<0.05. Scale bar: 25 µm.

Neuroinflammation, indicated by the presence of activated microglia, is a pathological hallmark of many neurodegenerative diseases (Lopez-Valdes and Martínez-Coria, 2016) and may indicate degeneration, dysfunction or remodelling of neurons. To determine whether the intraocular injection or transgene expression triggered alterations in microglia, microglia were immunolabelled with Iba1 (also known as AIF1) in cross-sectioned retinas (Fig. 3A). Cell bodies of Iba1-positive microglia were found in the ganglion cell layer (GCL), inner nuclear layer (INL) and outer nuclear layer (ONL) for all treatment groups as expected (arrowheads, Fig. 3A). In treated retinas, processes of Iba1-positive microglia were found throughout the plexiform layers, and this was more pronounced in hTDP-ΔNLS-GFP. Accordingly, the percentage area occupied by Iba1 immunoreactivity across all layers of the retina demonstrated significant increases (*P*<0.05) in the amount of labelling in hTDP-ΔNLS-GFP retinas, but not hTDP-WT-GFP and vehicle retinas (Fig. 3C). Additionally, glial fibrillary acidic protein (GFAP)-labelled astrocytes were examined (Fig. 3B), which demonstrated labelling restricted to the GCL in all three treatments. No significant changes in percentage area labelled or overall intensity were observed (Fig. 3D). These data indicate that the expression of cytoplasmic TDP-43 in RGCs is associated with an inflammatory response suggestive of neurodegenerative processes.

### Altered TDP-43 expression does not result in cell loss

**Fig. 4.**
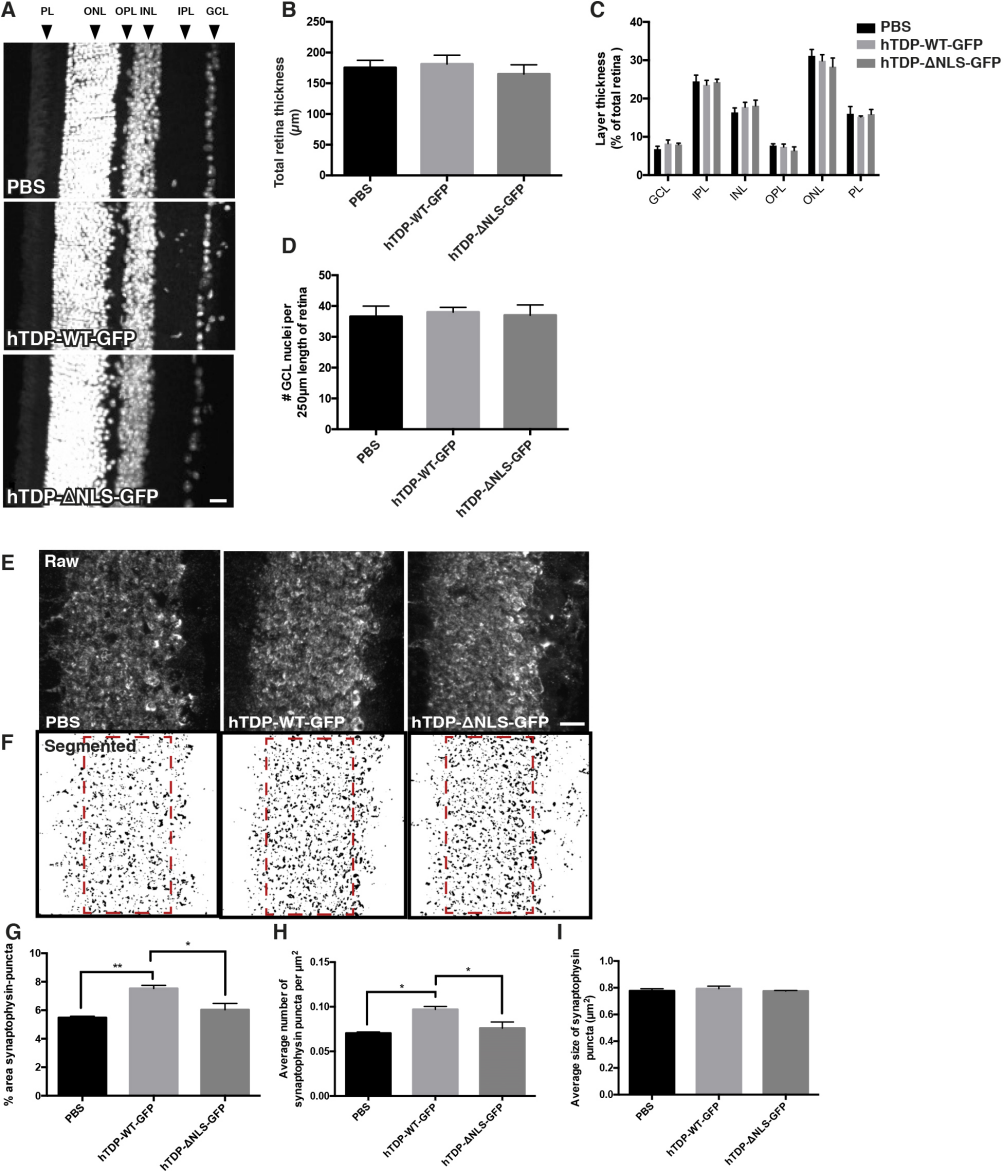
**Retinal cell number and synaptic changes associated with transduction with altered TDP-43.** (A) Representative images of DAPI-stained sectioned retina from mice injected with vehicle (PBS), hTDP-WT-GFP or hTDP-ΔNLS-GFP, showing retinal layers: ganglion cell layer (GCL), inner plexiform layer (IPL), inner nuclear layer (INL), outer plexiform layer (OPL), outer nuclear layer (ONL) and photoreceptor layer (PL). (B,C) Total retinal thickness (B) and thickness of each layer with respect to total thickness (C) were quantified. (D) The number of GCL nuclei stained with DAPI along the length of retina was quantified. (E) Representative images of sectioned retina from mice treated as above, immunolabelled with synaptophysin within the IPL. (F) Segmented synaptophysin images with ROIs indicated by red dashed line boxes. (G-I) Quantitation of segmented puncta per 3000 µm^2^ ROI yielded measurements of percentage area (G), synaptic density (H) and average size (I) of synaptophysin-positive synaptic boutons. Values are mean±s.e.m., *n*=5 per treatment group. *P*-values are from one-way ANOVAs with Tukey post hoc tests; *P<0.05, **P<0.01. Scale bars: 20 µm (A), 25 µm (E).

We next determined whether expression of TDP-43 constructs resulted in any gross cellular loss or changes in connectivity between the retinal layers. Retinal thinning occurs in some neurodegenerative diseases of the eye (Garcia-Martin et al., 2014; Abegg et al., 2014; Syc et al., 2012), and may indicate either cell loss or loss of axons or dendrites. In cross-sectioned retinas, the total thickness of the retina was measured and no differences were detected (Fig. 4A,B). Next, the thickness of each 4′,6-diamidino-2-phenylindole (DAPI)-stained layer with respect to the total thickness was determined (Fig. 4A,C), and, similarly, no differences were detected. To determine whether cells within the GCL layer (RGCs and displaced amacrine cells; Jeon et al., 1998) were lost, the number of nuclei in the GCL was counted from DAPI-stained sections. Neither the expression of hTDP-WT-GFP nor hTDP-ΔNLS-GFP altered the number of GCL nuclei relative to that in PBS vehicle-treated eyes (Fig. 4D).

### Synaptic density in the retina is increased in hTDP-WT-GFP-treated mice

To further determine the effect of TDP-43 expression on the connectivity between transduced RGCs and with cells synapsing onto these cells, pre-synaptic inputs from bipolar and amacrine cells to RGCs were immunolabelled with synaptophysin and quantitated in the inner plexiform layer (IPL) (Fig. 4E,F). There was a significant increase in both the percentage area labelled (*P*<0.05) and density (*P*<0.001) of synaptophysin-immunoreactive puncta in the IPL of hTDP-WT-GFP mice compared to PBS vehicle- and hTDP-ΔNLS-GFP-treated mice (Fig. 4G,H). There were no alterations to the average bouton size (Fig. 4I). This suggests that increased expression of nuclear TDP-43 has subtle effects on neuronal circuitry.

### Neurofilament localization is altered in hTDP-ΔNLS-GFP retinas

**Fig. 5.**
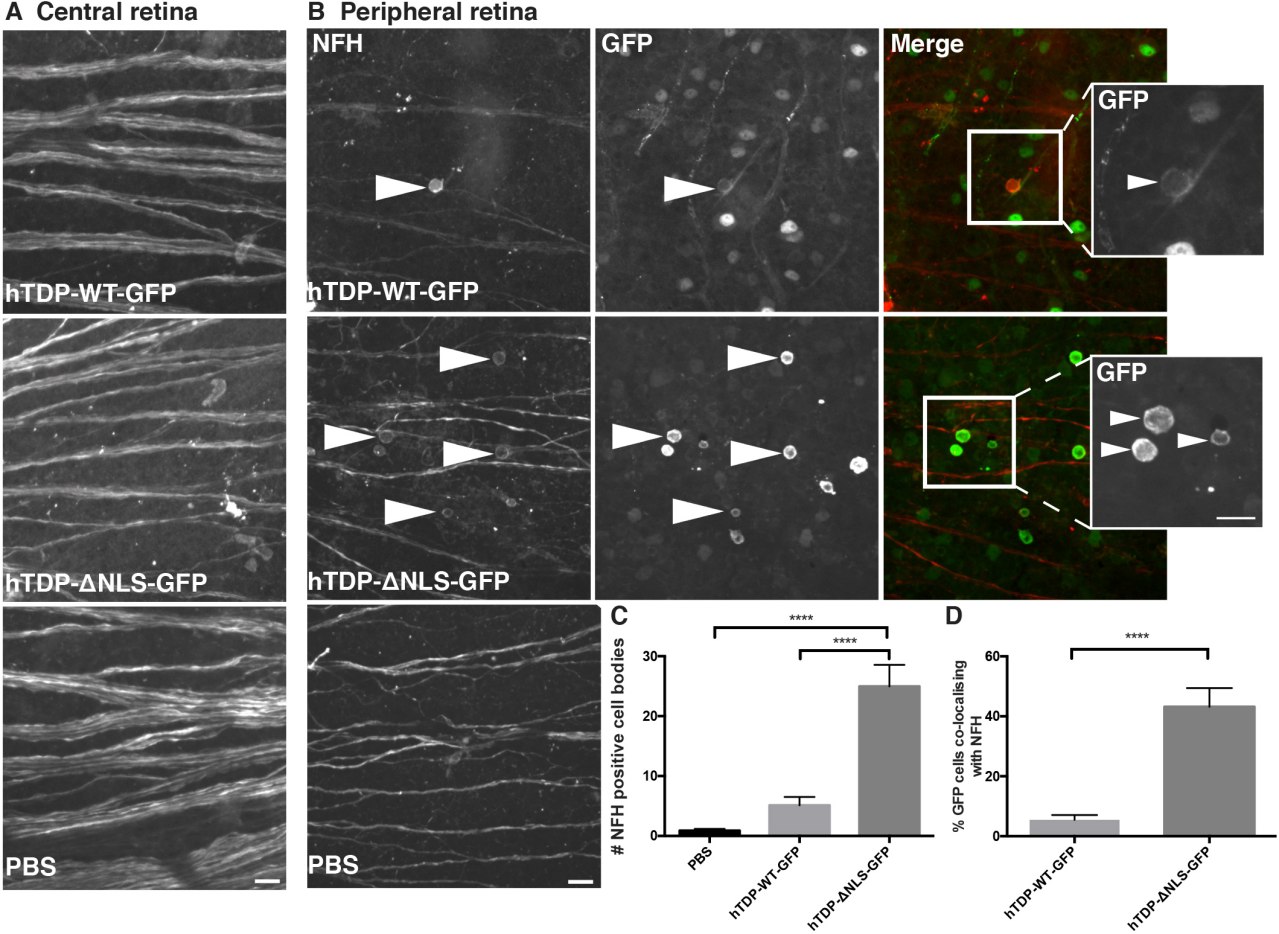
**Neurofilament heavy expression is mislocalized following TDP-43 alterations.** (A) Representative images taken close to the optic disc (central retina) of wholemount retina from mice injected with vehicle (PBS), hTDP-WT-GFP or hTDP-ΔNLS-GFP immunolabelled for neurofilament heavy (NFH). (B) Representative images taken in the peripheral portions of wholemount retina from mice treated as above, immunolabelled for NFH (red) or GFP (green). Arrowheads show increased NFH labelling in cell bodies, which also have GFP labelling in hTDP-WT-GFP- or hTDP-ΔNLS-GFP-injected tissue, demonstrating more perinuclear expression. Boxed areas are shown as magnified GFP images on the right. (C) The number of NFH-positive cell bodies in retinas from mice treated as above was quantified. (D) The percentage of GFP cells that had colocalizing NFH cell body immunolabelling was quantified in mice treated with hTDP-WT-GFP and hTDP-ΔNLS-GFP. Values are mean±s.e.m., *n*=5 per treatment group. *P*-values are from a two-tailed unpaired parametric Student's *t*-test; ****P<0.0001. Scale bars: 20 µm.

RGCs extend a single axon along the surface of the retina to the optic disc, where it enters the optic nerve and becomes myelinated. Neurofilament immunolabelling in RGC axons was qualitatively examined for morphological changes relative to the expression of altered TDP-43. Wholemount retinas were labelled with a polyclonal antibody to the neurofilament heavy subunit (NFH; phosphorylation independent). In central parts of the retina (close to the optic disc), there was strong, continuous NFH immunolabelling of axon bundles, with no qualitative differences between treatment groups (Fig. 5A). In more peripheral portions of the retina from all treatment groups, NFH labelling continued to be strongly expressed in axons (Fig. 5B). However, there was a significant increase in the proportion of cells that expressed NFH in the cell body in hTDP-ΔNLS-GFP retinas compared to both PBS vehicle and hTDP-WT-GFP retinas (*P*<0.0001; Fig. 5C). In hTDP-ΔNLS-GFP tissue, NFH immunoreactivity colocalized with 43.2±6.3% of GFP-positive cell bodies, whereas in hTDP-WT-GFP tissue only 5.45±1.64% of GFP-positive cell bodies were labelled for NFH (*P*<0.0001; Fig. 5D). NFH was rarely present in cell bodies that were not GFP positive.

### Neurofilament expression is altered in optic nerves of treated mice

**Fig. 6.**
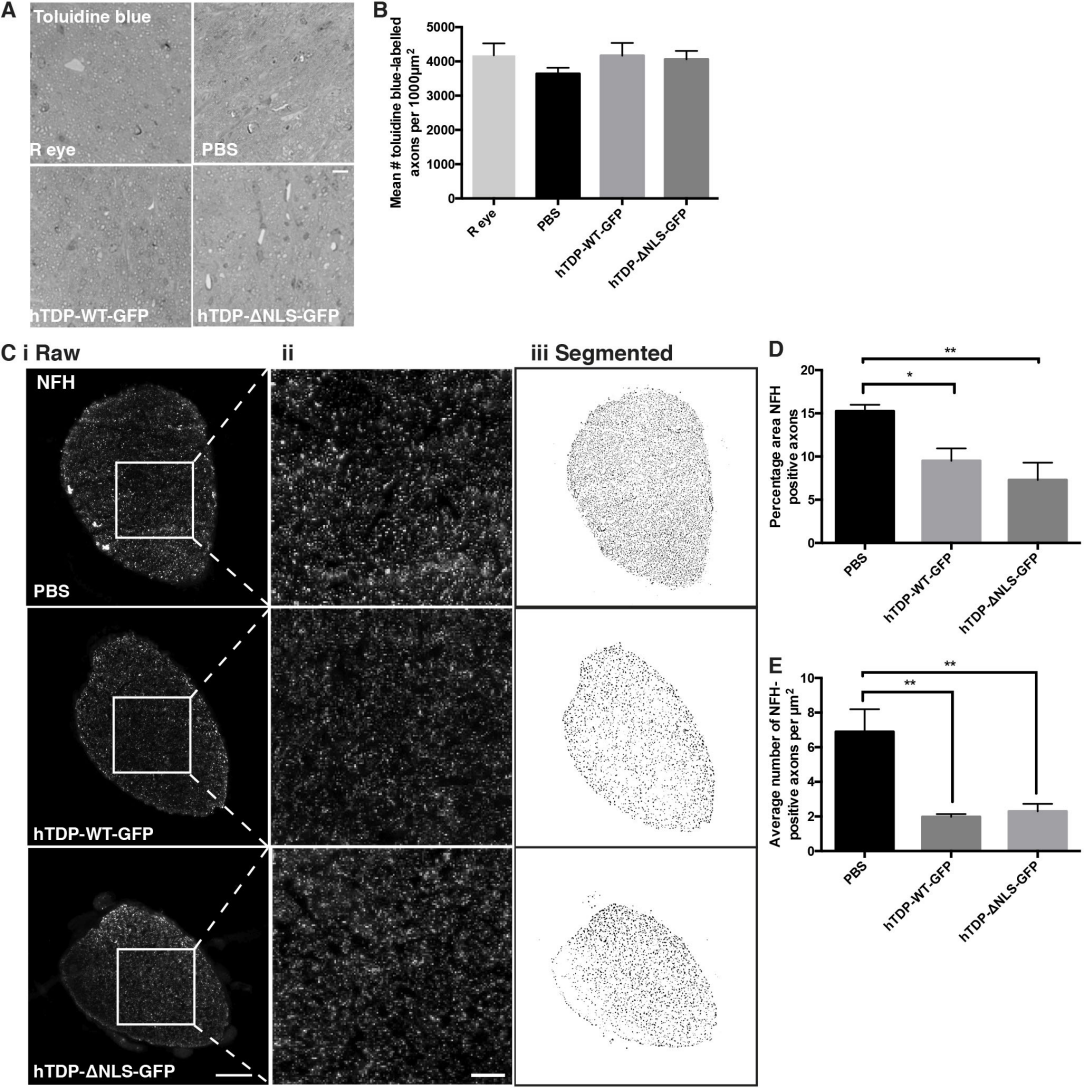
**NFH immunolabelling is reduced in optic nerve axons following TDP-43 alterations.** (A) Representative images of Toluidine Blue-stained semi-thin optic nerve cross sections from uninjected eye (R eye), and mice injected with vehicle (PBS), hTDP-WT-GFP or hTDP-ΔNLS-GFP. (B) Quantitation of 1000 µm^2^ ROI within Toluidine Blue-stained sections yielded measurements of mean number of axons per ROI, *n*=3 per treatment group. (C) Cross-sectioned optic nerves from mice injected with vehicle (PBS), hTDP-WT-GFP or hTDP-ΔNLS-GFP immunolabelled for NFH. Column i shows original raw NFH-labelled images; column ii shows magnified images of the boxed areas in column i; and column iii shows segmented images. (D,E) Segmented puncta per whole optic nerve yielded measurements of percentage area occupied by NFH-positive axons (D) and average number of NFH-positive axons per optic nerve (E), *n*=5 per treatment group. Values are mean±s.e.m., *n*=5 per treatment group. *P*-values are from one-way ANOVAs with Tukey post hoc tests; *P<0.05, **P<0.01. Scale bars: 10 µm (A), 50 µm (C,i), 10 µm (C,ii).

We next examined whether altered TDP-43 expression had effects on RGC axons in the optic nerve. Toluidine Blue-stained semi-thin sections showed no difference in the number of axons per 1000 µm^2^ area (Fig. 6A,B). However immunolabelled cryosections showed that the percentage area of the optic nerve occupied by NFH-positive axons was significantly (*P*<0.05) reduced in both hTDP-WT-GFP and hTDP-ΔNLS-GFP optic nerves compared to PBS vehicle optic nerves (Fig. 6C,D), with a corresponding significant (*P*<0.05) decrease in the number of NFH-positive axons (Fig. 6E).

We then looked to see whether these changes were accompanied by any indicators of degeneration or dysfunction in longitudinally sectioned optic nerves. There was no indication of dephosphorylated neurofilament (SMI32 immunolabelling) or alpha internexin accumulation in the optic nerves from any of the treatments (Fig. S3).

### Alteration to TDP-43 expression is not associated with ultrastructural alterations to RGC axons

**Fig. 7.**
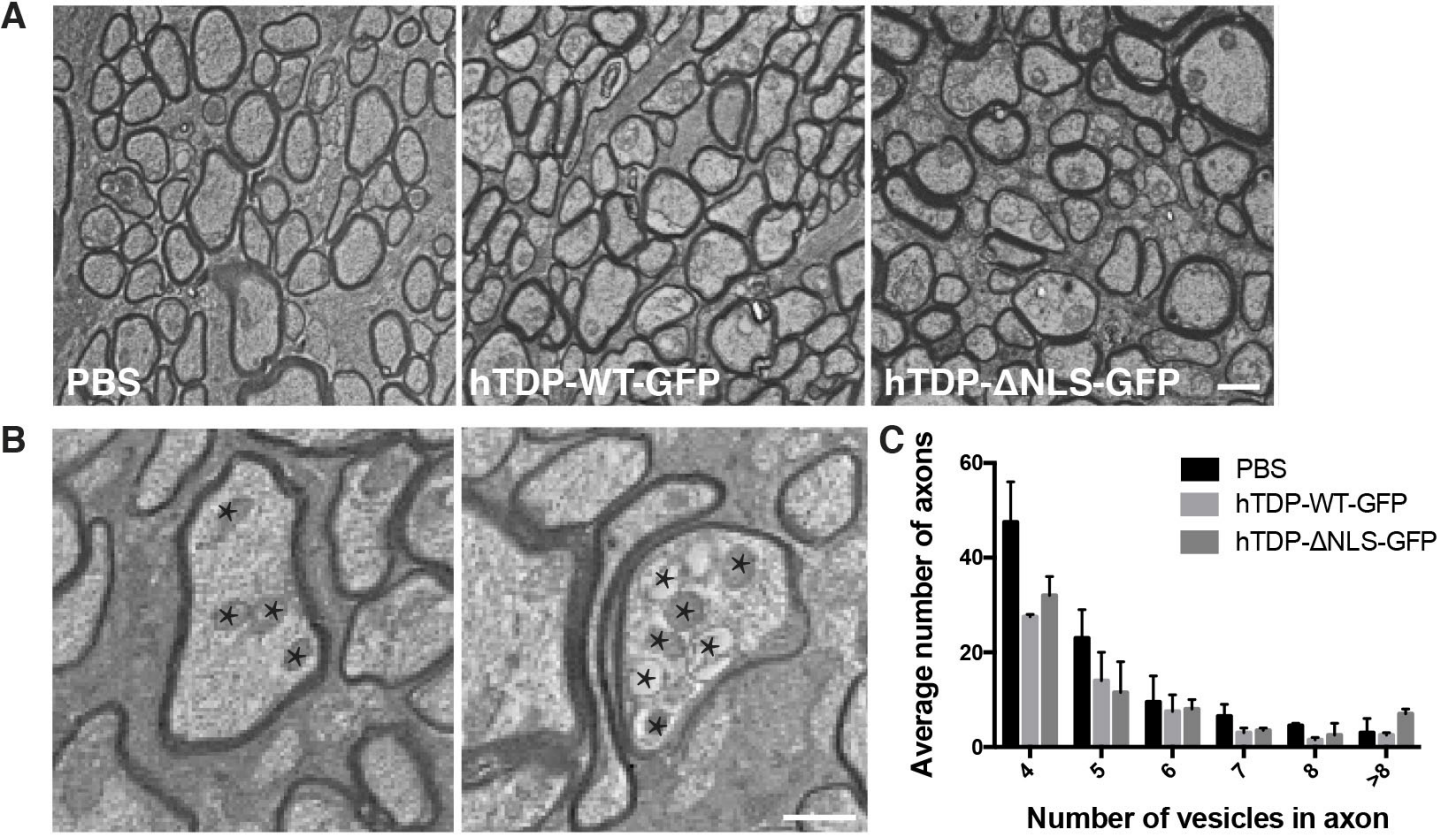
**Ultrastructural changes in cross-sectioned optic nerve.** (A) Representative electron micrographs demonstrating axonal profiles from mice injected with vehicle (PBS), hTDP-WT-GFP or hTDP-ΔNLS-GFP. (B) Example images of axons containing vesicles (indicated by stars). (C) The numbers of axons with differing numbers of vesicles were quantified. Values are mean±s.e.m., *n*=3 per treatment group. Scale bars: 1 µm.

In order to determine whether alterations to TDP-43 caused ultrastructural changes to RGC axons, analysis of optic nerves was carried out using transmission electron microscopy (TEM; Fig. 7A). Optic nerves were cross sectioned proximal to the optic nerve head, and then also sectioned 5 µm distal to this to examine changes along axons. Qualitatively, axons from all treatment groups appeared similar. Close examination of axon morphology identified a subset characterized by accumulation of both normal and dysmorphic organelles (examples shown in Fig. 7B), which can indicate alterations to transport processes. Axons containing different numbers of vesicles were quantitated (Fig. 7C). The distribution of axons with differing numbers of vesicles was relatively even amongst treatment groups, with PBS treatment resulting in higher numbers of axons containing four and five vesicles, and hTDP-ΔNLS-GFP treatment resulting in higher numbers of axons with more than eight vesicles. However, axons containing greater than eight vesicles were observed in all treatment groups.

### TDP-43 alterations do not lead to RGC pre-synaptic terminal changes

**Fig. 8.**
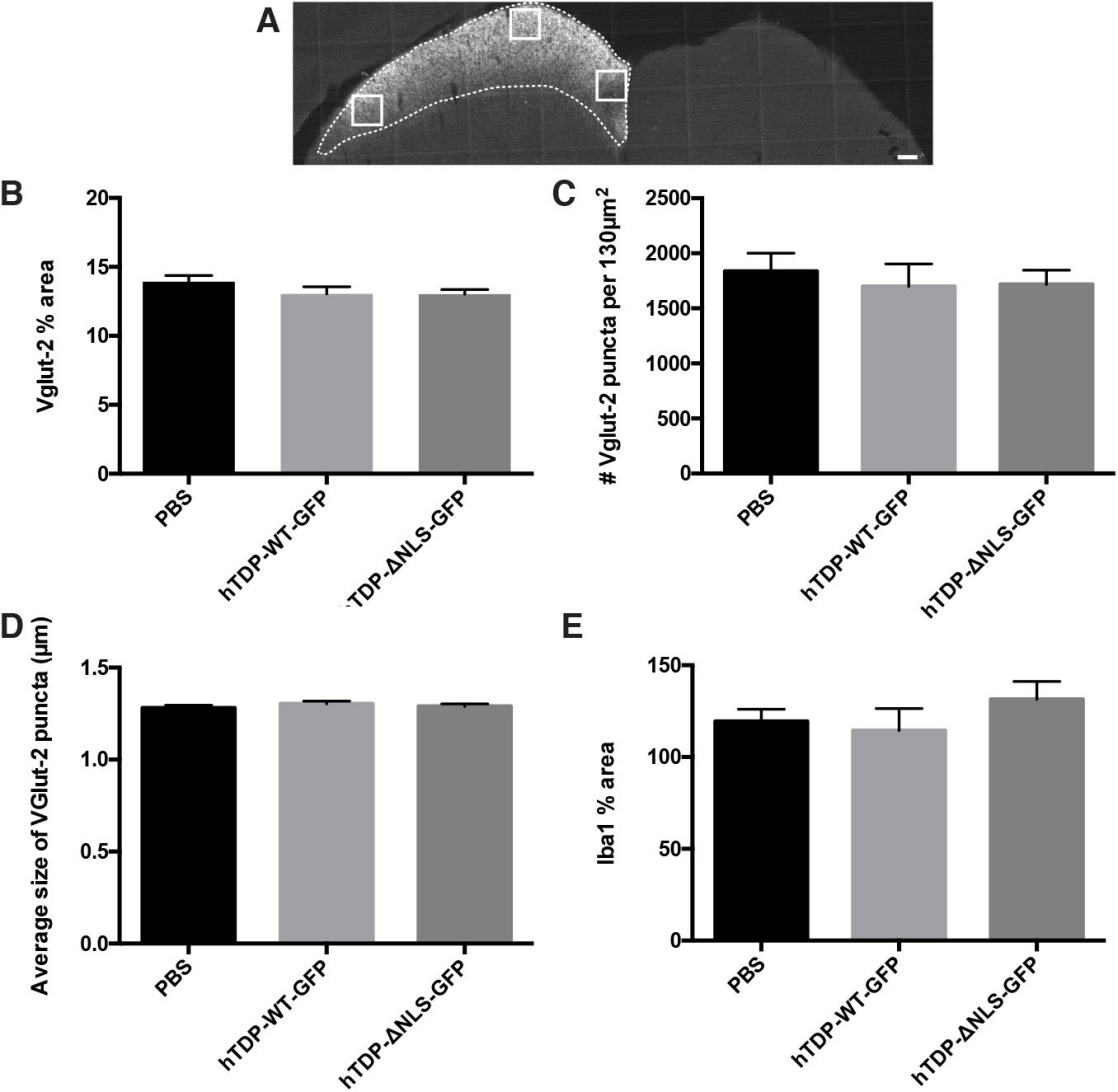
**TDP-43 alterations do not cause RGC pre-synaptic changes.** (A) Representative image of coronally sectioned brain showing RGC terminals within the SC following intraocular injection with choleratoxin subunit b (CTB). White boxes indicate ROIs used for Vglut2 analysis, and dotted white lines indicate the edge of CTB labelling, used for Iba1 analysis. Mice were injected with vehicle (PBS), hTDP-WT-GFP or hTDP-ΔNLS-GFP. (B-D) Quantitation of segmented puncta pooled from three ROIs immunolabelled with Vglut2 yielded measurements of percentage area (B), average number (C) and average size (D) of Vglut2-positive synaptic boutons. (E) Quantitation of segmented microglia immunolabelled with Iba-1 within CTB-labelled SC yielded the percentage area occupied by Iba1-positive microglia, when normalized to the same ROI on the contralateral SC. Values are mean±s.e.m., *n*=5 per treatment group. Data were analysed with one-way ANOVAs with Tukey post hoc tests, showing no significant differences. Scale bar: 150 µm.

In mice, the SC is a primary termination site of RGC axons (May, 2006), where alterations in presynaptic terminals may indicate changes in RGC axons. The density of synaptic terminals labelled with Vglut2 (also known as SLC17A6) (Hammer et al., 2014) were analysed within three regions (Fig. 8A) of the SC identified as RGC terminals by choleratoxin subunit b (CTB) labelling. There were no significant changes between treatments in the percentage area occupied by Vglut2-positive puncta, average size of Vglut2-positive puncta or the number of Vglut2-positive puncta (Fig. 8B-D). Synaptic degeneration and loss is mediated, in part, by microglia. Therefore, to further examine whether subtle changes were occurring, prior to overt loss of synapses in the SC, tissue was immunolabelled with an antibody against Iba1 and examined within the CTB-labelled portion of the SC (Fig. 8A). When normalized to Iba1 immunolabelling in the contralateral SC, there were no significant changes in the percentage area of Iba1 immunolabelling between treatment groups (Fig. 8E). Together, these results indicate that there were no overt pre-synaptic changes to RGC terminals in the SC following TDP-43 alteration.

## DISCUSSION

**Fig. 9.**
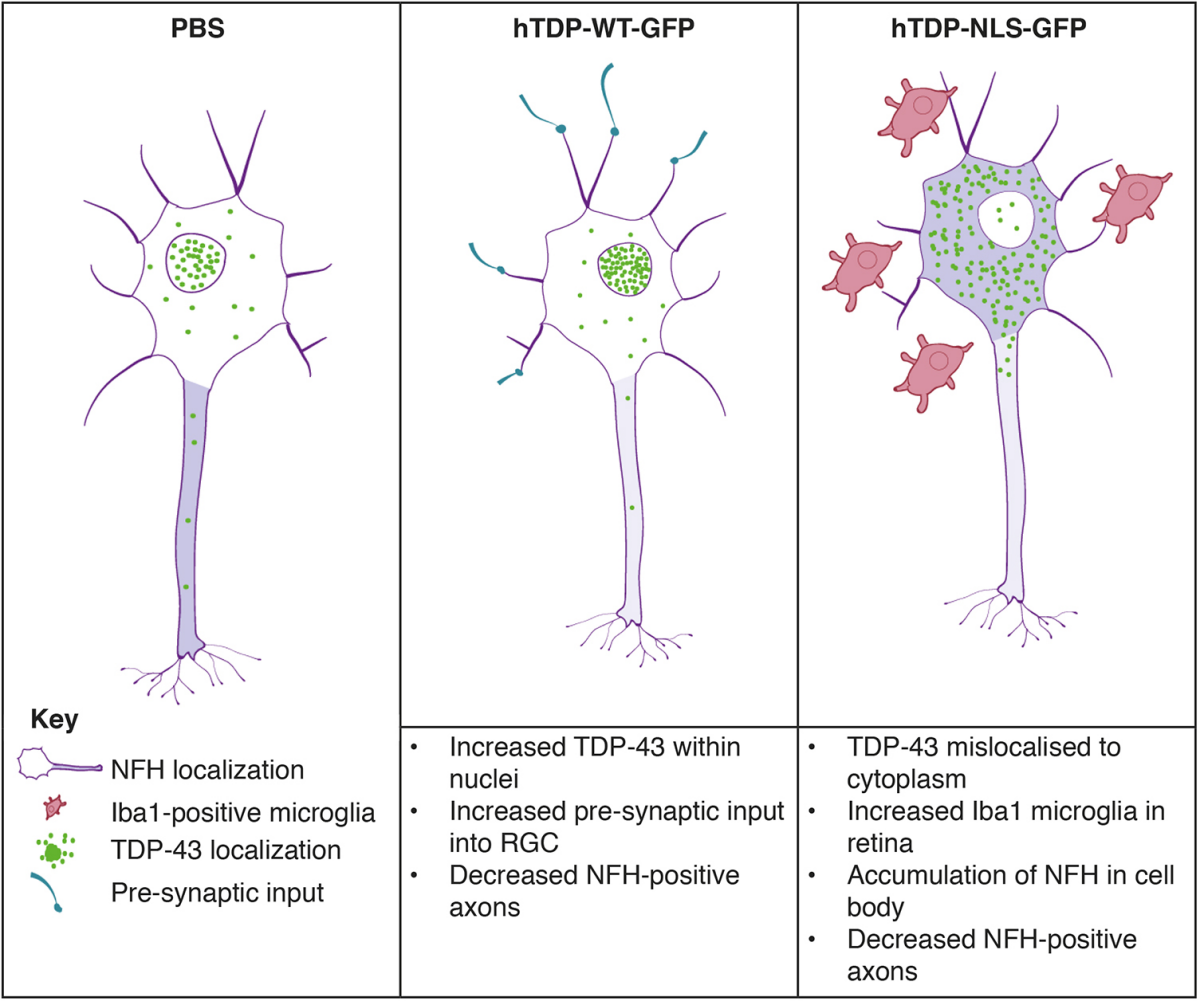
**Schematic illustrating the main changes to neurons following treatment with PBS, hTDP-WT-GFP or hTDP-ΔNLS-GFP.**

Mislocalization of TDP-43 from the nucleus to the cytoplasm has been proposed to be an important driver of cellular dysfunction in FTLD and ALS. This study has established a new *in vivo* model for examining whether alterations to the expression level and localization of TDP-43 have downstream effects on neuronal morphology, connectivity and health. This experimental approach provides a novel system to study CNS neuron function, synaptic and morphological changes in the retinal layers, alterations to axons as they enter the optic nerve, and connectivity within the brain. High transduction efficiency rates for both hTDP-WT-GFP and hTDP-ΔNLS-GFP under the CAG promoter were demonstrated, allowing downstream changes in the optic nerve to be more reliably attributed to overexpression of the transgene. We showed that altered TDP-43 resulted in changes to neurofilament proteins in RGCs; cytoplasmic TDP-43 (hTDP-ΔNLS-GFP) resulted in localization of neurofilament (NFH) to the cell soma, which was associated with increased numbers of microglia. Furthermore, both increased nuclear (hTDP-WT-GFP) and cytoplasmic TDP-43 resulted in reduced numbers of NFH-positive axons. Expression of hTDP-WT-GFP was also associated with an increase in pre-synaptic input into RGCs. These findings are summarized in Fig. 9.

RGCs transmit visual information from the outer retina layers to the visual areas of the brain. To assess visual function following RGC transduction, the well-established optomotor response test of visual acuity was used (Abdeljalil et al., 2005). RGC loss has been shown to reduce acuity (Ellouze et al., 2008). However, in the current study, there was no significant effect of treatment on this visual parameter, despite alterations to RGCs observed in hTDP-WT-GFP- and hTDP-ΔNLS-GFP-treated mice.

This study modelled both overexpression of WT hTDP-43 and mislocalization of hTDP-43 to the cytoplasm, both of which may be associated with disease processes in ALS/FTD. Eyes injected with hTDP-WT-GFP demonstrated nuclear localization within RGCs, with no abnormal mislocalization to the cytoplasm, and which was free from GFP-positive aggregates. Multiple studies suggest that mislocalization of overexpressed WT hTDP-43 depends on the rate of overexpression. In cell culture studies by Barmada et al. (2010), transfection with low levels of WT hTDP-43 did not result in cytoplasmic localization, and there was no effect on cell survival, whereas higher concentrations caused variable mislocalization to the cytoplasm and a reduction in cell survival (Barmada et al., 2010). Similarly, overexpressing WT hTDP-43 on the mouse prion promoter in mice, at a rate 2.5× endogenous mTDP-43 expression, produced cytoplasmic mislocalization accompanied by severe neurodegeneration (Xu et al., 2010). By contrast, overexpression of TDP-43 at both 1.5× (Arnold et al., 2013) and 1.9× (Xu et al., 2010) the endogenous rate resulted in phenotypically normal mice. A limitation of the current study was that the exact rate of overexpression was not known. It is possible that the level of hTDP-WT-GFP expression and/or the length of the experiment were insufficient to allow observation of overt degeneration. Additionally, it would be interesting to examine the effects of GFP expression alone. It is known that GFP expression can lead to some cellular effects such as oxidative stress (Ganini et al., 2017). However, this control group would also have its limitations, with GFP being expressed throughout the entire neuron and not only in the areas where WT or NLS TDP-43 were located.

Expression of hTDP-WT-GFP led to an increase in the number and percentage of synaptophysin-positive synapses in the IPL of the retina, which contains cell processes and synapses between both amacrine and bipolar cells and the RGCs. Although we were unable to effectively label post-synapses of RGC in the IPL in the current study, these results are in line with previous findings that alterations to TDP-43 lead to changes in connectivity. For example, overexpression of WT TDP-43 in *Drosophila* led to an increase in dendritic branching of sensory neurons (Lu et al., 2009), and alterations to dendritic branching likely result in changes to neuronal connectivity as seen in human studies (Seeley et al., 2009). We also examined the impact of TDP-43 overexpression on RGC pre-synapses within the SC but found no differences between treatments. Further studies are needed to more carefully define the impact of increased expression of nuclear TDP-43 on post-synaptic connectivity of neurons.

In contrast to hTDP-WT-GFP-transduced retinas, transduction of RGCs with hTDP-ΔNLS-GFP resulted in strong GFP fluorescence in the cytoplasm and the primary neurites of a subset of cells. Cytoplasmic TDP-43 has specific roles separate to roles in the nucleus. Previous research investigating TDP-ΔNLS constructs in cultured neurons has demonstrated high neuritic expression (Barmada et al., 2010) and axonal accumulation of exogenous TDP-43 (Winton et al., 2008), which has also been recapitulated *in vivo* (Tripathi et al., 2014). A key finding of the current study was that cytoplasmic TDP-43 was associated with mislocalization of NFH to the cell bodies, with a small subset of hTDP-WT-GFP-transduced neurons also exhibiting this change. ALS is characterized by filamentous aggregates of neurofilament in the perikaryon, proximal and distal axons (Leigh and Swash, 1991; Cifuentes-Diaz et al., 2002; Liu et al., 2004), and it has been demonstrated that neurofilament mRNA is reduced in ALS (Wong et al., 2000; Menzies et al., 2002). Similar alterations to neurofilament proteins are one of the earliest changes in transgenic mouse models of ALS (Tu et al., 1996; Morrison et al., 2000), and alterations to neurofilament proteins are also a frequent finding in other neurodegenerative diseases and following injury; for example, neurons demonstrate decreased expression of neurofilament proteins in the cell body after axotomy (Mikucki and Oblinger, 1991), and, after nerve crush injury, phosphorylated epitopes of neurofilaments, normally localized to axons, can be observed in the cell body (Moss and Lewkowicz, 1983).

Neurofilament proteins are obligate heteropolymers composed of neurofilament light, medium and heavy chains as well as alpha internexin and/or peripherin. Changes to NFL expression (either overexpression or knockout) can lead to altered stoichiometry of other subunits including NFH (Liu et al., 2013) or can cause a buildup of neurofilament proteins within the soma (Liu et al., 2013; Xu et al., 1993; Julien et al., 1995). Thus, although there is tight topographical regulation of expression and phosphorylation of neurofilament proteins, abnormal accumulation in the cell body as observed in the current study may occur if filament assembly is abnormal. TDP-43 has been shown to be a modulator of *Nefl* (encoding NFL) mRNA (Strong et al., 2007). In the nucleus TDP-43 binds to the 3′UTR of *Nefl* mRNA, stabilizing it within the nucleus and regulating its translocation to the cytosol, where it may also regulate its translation (Strong et al., 2007; Volkening et al., 2009). However, cytoplasmic TDP-43 may recruit *Nefl* mRNA to stress granules, thus reducing its expression (Strong et al., 2007). This may suggest that altered expression of NFL induced by mislocalization of TDP-43 could drive altered stoichiometry of other neurofilament proteins in our model and drive the somal accumulation that we have demonstrated. Alternatively, phosphorylation of the N-terminus of neurofilaments by protein kinase A and C governs their localization to the soma or axon (Yates et al., 2009), and a dysfunction of this process could potentially occur following transduction with hTDP-ΔNLS-GFP. Further studies would be required to determine this.

Although there were no differences in the total number of axons within treated optic nerves, suggesting that axons were not being lost, there was a decrease in the number and percentage area of NFH-positive axons in the optic nerves of mice treated with both hTDP-WT-GFP and hTDP-ΔNLS-GFP, which occurred without alterations to neurofilament phosphorylation (SMI32 immunoreactivity). This again indicates a link between TDP-43 and neurofilament proteins and may suggest that nuclear TDP-43 and cytoplasmic TDP-43 drive different changes in neurofilament expression, both of which can result in decreased distal axonal neurofilament expression.

There were changes to the density of microglia in hTDP-WT-GFP-transduced retinas. Inflammation is an important aspect of ALS and FTD, with alterations to microglia, the resident immune cells of the CNS, being a pathological hallmark of these diseases (Lopez-Valdes and Martínez-Coria, 2016; Piguet, 2013) and microglial activation occurring in areas of protein inclusion pathology in ALS (Brettschneider et al., 2012). Brettschneider et al. (2012) demonstrated microglial activation in brain regions with high TDP-43 pathology, and, in studies using mouse models with an inducible form of hTDP-ΔNLS-GFP, activated astrocytes and microglia are observed in the cortex and hippocampus within 1 month of expressing the transgene (Igaz et al., 2011). Microglial activation and migration is also observed following CNS injury (Kreutzberg, 1996). In light of the involvement of microglia in engulfment of synapses (Henstridge et al., 2019), it was interesting that an increase rather than a decrease in synaptophysin labelling was observed in the IPL of the retina. Future studies further investigating the interaction between synapses and microglia in normal TDP-43 function, and in TDP-43-induced degeneration, would be of interest.

Despite the alterations to neurofilament proteins demonstrated in our study, we did not observe severe axon degeneration, or evidence of ultrastructural changes to axons induced by expression of our constructs. Previous studies using transgenic mice demonstrate that manipulation of the TDP-43 NLS is sufficient to recapitulate some features of axon degeneration, with select loss of neurofilament-positive corticospinal tract axons (Igaz et al., 2011) and dieback of motor neuron axons from the neuromuscular junction (Walker et al., 2015). These alterations may indicate that there might be cell-type-specific responses to neurofilament alterations or that our model is related to a less-severe phenotype. Future studies examining longer-term effects of altered TDP-43 would be valuable in further teasing out these theories.

In summary, the current study has developed a new model allowing detailed examination of alterations caused by TDP-43 overexpression and mislocalization of TDP-43. This model provides a unique way to study alterations to the CNS following alterations to disease-related processes. In future studies, it will allow rapid comparison of the effects of different genetic mutations as well as screening of therapeutic agents and how these may modulate disease processes. This study has demonstrated that both nuclear and cytoplasmic TDP-43 can affect the expression or localization of neurofilament proteins. This could have consequences on interpreting pathological changes to neurofilament proteins in post-mortem tissue and mouse models of ALS and FTLD. These results will contribute to the knowledge of TDP-43-mediated neuronal alterations and degeneration.

## MATERIALS AND METHODS

### Animals

All experiments involving animals were approved by the University of Tasmania Animal Ethics Committee (A14189 and 16522) in accordance with the Australian Guidelines for the Care and Use of Animals for Scientific Purposes (National Health and Medical Research Council, 2013), and followed Animal Research: Reporting of *In Vivo* Experiments (ARRIVE) guidelines. Animals were housed in standard conditions (20°C, 12 h/12 h light/dark cycle), with access to food and water *ad libitum.* Male mice were used for this study.

### Generation of AAV2 viruses

Constructs contained human WT *TARDBP* (hTDP-WT; NM_007375) or human *TARDBP* with a mutated nuclear localization sequence (hTDP-ΔNLS), fused at the C-terminus to mGFP (Fig. 1C). A commercial lentiviral TDP-43 plasmid (RC210639 *TARDBP* insert, cloned into pLenti-C-mGFP PS100071, Origene Technologies) was purchased, and site-directed mutagenesis of the human TDP-43 protein sequence was used to create a defective NLS through a missense mutation (ΔNLS1, K82A/R83A/K84A), as described in Winton et al. (2008). GFP-tagged TDP-43 inserts (hTDP-WT-GFP and hTDP-ΔNLS-GFP) were cloned in AAV plasmids under the control of the CAG hybrid promoter, consisting of the cytomegalovirus and chicken β-actin promoter (Martin et al., 2002) and packaged into AAV serotype 2 (AAV2) virus particles by Vector Biolabs (Malvern, PA, USA). The hTDP-WT-GFP virus had a titre of 2.7×10^13^ GC/ml and hTDP-ΔNLS-GFP virus had a titre of 1.2×10^13^ GC/ml, determined by quantitative PCR.

### Determination of optimal viral titre

#### Eye injections

Mice were anaesthetized in an induction chamber filled with 5% isoflurane prior to intravitreal injection. The mice were maintained under anaesthetic throughout the surgery procedures with 2.5% isoflurane at a flow rate of 0.8 l/min. A hole was first created in the temporal quadrant of the sclera of the left eye with a 31-gauge needle, and 1 µl PBS or virus was slowly injected into the vitreous humour using a 33-gauge needle connected to a Nanofil syringe (intraocular injection kit, World Precision Instruments) (Massoll et al., 2013). The needle tip was inserted into the superior hemisphere of the eye at the level of the pars plana, at a 45° angle through the sclera and into the vitreous body. This route of administration was used to avoid retinal detachment or injury to the lens and iris. The injection was performed over a timecourse of 10 s, after which the needle was held in place for an additional 30 s to prevent leakage of the virus/vehicle. Following this, the mouse was allowed to recover on a heat pad.

To determine the optimal injection titre of virus, a pilot study with 12 C57Bl/6J mice (aged 10-16 weeks, *n*=12) were injected with 1 µl serially diluted hTDP-WT-GFP in PBS (nine dilution series starting at 1.35×10^13^) and left for 1 month for the virus to be expressed. An additional study was carried out using hTDP-ΔNLS-GFP AAV2, with mice perfused at 7 (*n*=2), 14 (*n*=2) and 28 (*n*=2) days to determine when viral induction could be observed.

In mice, synaptogenesis occurs within the first to third week of postnatal development (Pfrieger, 2009), then synapses are pruned to adult levels at ∼4 months of age (De Felipe et al., 1997). To ensure synaptic maturity, 6-month-old C57Bl/6J mice were used. Treatment groups included vehicle (PBS, *n*=9), hTDP-WT-GFP (*n*=9), hTDP-ΔNLS-GFP (*n*=9), with five animals used for downstream immunohistochemical analysis and four animals used for TEM. Group allocation was blinded prior to commencement of the study. Mice were randomly allocated to each treatment group, ensuring that a mixture of treatments was included in each mouse cage. The number of mice required for downstream immunohistochemical analyses was based on the ‘Resource Equation’, where *n*=(*E*+*T*)/*T* (Charan and Kantharia, 2013). *n* represents the number of animals per treatment group. *E* represents the degrees of freedom of the analysis of variance (ANOVA), and should lie between 10 and 20; in this case, it is equal to 12. *T* is the number of treatments, in this case 3 (vehicle, hTDP-WT-GFP and hTDP-ΔNLS-GFP). Based on these numbers, *n*=5. Mice were anaesthetized in an induction chamber filled with 5% isoflurane prior intravitreal injection. The mice were maintained under anaesthetic throughout the surgery procedures with 2.5% isoflurane at a flow rate of 0.8 l/min. A hole was first created in the temporal quadrant of the sclera of the left eye with a 31-gauge needle, and 1 µl PBS or virus at optical concentration (determined in a pilot study) was slowly injected into the vitreous humour using a 33-gauge needle connected to a Nanofil syringe (intraocular injection kit, World Precision Instruments) (Massoll et al., 2013). The needle tip was inserted into the superior hemisphere of the eye at the level of the pars plana, at a 45° angle through the sclera and into the vitreous body. This route of administration was used to avoid retinal detachment or injury to the lens and iris. The injection was performed over a timecourse of 10 s, after which the needle was held in place for an additional 30 s to prevent leakage of the virus/vehicle. Following this, the mouse was allowed to recover on a heat pad.

Mice were left for 3 months to allow expression of the constructs and development of pathology, based on evidence that an inducible TDP-43 mouse model overexpressing the same NLS defect developed axonal changes 4 weeks post-transgene induction and had severe denervation of neuromuscular junctions by 6 weeks post-induction (Walker et al., 2015). For downstream analysis of the SC, an additional cohort of animals (*n*=5 per treatment group) was injected with TDP-WT, TDP-NLS and PBS and left for 3 months as above, but received an intravitreal injection of 1 µl CTB (1%; Invitrogen, Molecular Probes) 2 days prior to perfusion.

#### Optomotor response

The optomotor response was used to measure alterations to visual acuity in mice injected with TDP-43 constructs or vehicle and was measured as previously described (Heitz et al., 2012) based on principles described by Abdeljalil et al. (2005). The optomotor response (defined as head turning to match the speed of drum rotation) was tested by rotating the drum clockwise or anticlockwise. Testing was carried out 1 week pre-injection, 1 week post-injection and then fortnightly until experiment endpoint (3 months). In mice, the left (treated) eye controls the clockwise optomotor response, and the right (untreated) eye controls the anticlockwise response (Heitz et al., 2012).

#### Tissue processing

At 9 months of age, 3 months post-treatment, animals were sacrificed for immunohistochemistry (*n*=5 animals/treatment, as described above; *n*=5 animals/treatment for CTB studies) or TEM (*n*=4 animals/treatment). Transcardial perfusion was carried out with 4% paraformaldehyde (Sigma-Aldrich) in 0.1 M PBS (for immunohistochemistry) or 0.9% saline followed by 60 ml of 2% paraformaldehyde and 2.5% glutaraldehyde (Electron Microscopy Sciences) in 0.1 M phosphate buffer for TEM. All tissue was post-fixed in perfusion solution at 4°C overnight. The following day, tissue was dissected for immunohistochemistry to obtain retinas (divided for wholemount/flatmount, and cross sectioning), optic nerves (proximal portion for cross sectioning, distal portion for longitudinal sectioning) and brain, and stored at 4°C in 0.2% sodium azide in PBS. For TEM, the proximal half of the optic nerve was dissected for TEM and stored in 0.1 M phosphate buffer.

#### Immunohistochemistry

Tissue to be sectioned was cryoprotected in increasing concentrations of sucrose (18% then 30%; Liu et al., 2015). From each animal (*n*=5), three 16-µm-thick sections of retina (cross sections incorporating tissue from the peripheral to inner retina) and optic nerve (cross and longitudinal sections), spaced ∼128 µm apart, were obtained from sectioning tissue on a cryostat (Leica) and mounted directly onto slides (DAKO). Serial coronal sections (40 µm thick) were obtained from brains from Bregma positions −3.80 mm to −4.48 mm, which includes the SC (Franklin and Paxinos, 2008). Immunohistochemistry was carried out on samples individually blinded (not based on treatment groups) on free-floating sections, mounted slides or flatmount retina preparations as described previously (Atkinson et al., 2015). Flatmount retinas were incubated in primary antibody for 48 h to ensure optimal antibody penetration. Primary antibodies included anti-RBPMS (RNA-binding protein with multiple splicing, a marker of RGCs; Genetex, GTX118619, rabbit; 1:1000), anti-GFP (Invitrogen, A10262, chicken; 1:1000), anti-GFP (NeuroMab, 75-131, mouse; 1:1000), anti-GFAP (NeuroMab, G3893, mouse; 1:1000), anti-Iba1 (Wako, 019-19741, rabbit; 1:1000), anti-NFH (Millipore, AB5539, chicken; 1:1000), anti-synaptophysin (Millipore, AB9272, rabbit; 1:500), anti-Vglut2 (Synaptic Systems, 135421, mouse; 1:1000), anti-SMI32 (Biolegend, 801701, mouse; 1:1000), anti-alpha-internexin (Novus Biologicals, NB300-139, rabbit; 1:1000) and anti-phosphorylated TDP-43 (PS409/410, Cosmobio, TIP-PTD-P02, rabbit; 1:1000).

### TEM sample preparation

#### Fixation

Secondary fixation in 1% osmium tetroxide (Electron Microscopy Sciences) was carried out for 2 h at room temperature, followed by two washes at room temperature in 0.1 M phosphate buffer and a final wash in 4°C 0.1 M phosphate buffer. Samples were then washed twice with deionized H_2_O (dH_2_O), and tertiary fixation was carried out with 1% uranyl acetate (Merck) for 30 min at room temperature. Samples were washed three times in dH_2_O and dehydrated through graded acetone solutions (70% 1×8 min; 90% 1×8 min; 95% 1×8 min; 100% 4×5 min).

#### Embedding

Samples were incubated in propylene oxide (ProSciTech, 2×5 min) and embedded in resin. Embedding consisted of a 2 h incubation in 1:1 mix of propylene oxide and resin (Procure 812, ProSciTech); 10 min incubation at 60°C in resin, followed by overnight incubation at room temperature in the dark in resin; and finally embedding in resin at 60°C for at least 24 h to allow curing.

#### Sectioning and staining

Semi-thin sections (350 µm) were cut on a Reichert UltraS ultramicrotome and stained with Toluidine Blue (in 1% borax) for 30 s at 60°C, and rinsed with cold dH_2_O. Ultra-thin sections (70 nm) were collected onto copper grids and stored in a desiccator. Samples on grids were stained with uranyl acetate and lead citrate according to Reynolds (1963). Briefly, grids were incubated in saturated uranyl acetate solution (5% in 50% ethanol/water) in the dark for 30 min, washed in dH_2_O and incubated in Reynolds' lead citrate (Sigma-Aldrich) for 5 min at room temperature, in a CO_2_-depleted environment. Samples were washed with dH_2_O and stored in a desiccator.

#### Image acquisition and analysis

For each analysis, images were acquired with the same microscope settings. Imaging was carried out by a researcher blinded to treatment group. For analyses, images were separated into blinded treatment groups.

Transduction efficiency was calculated by manually counting and calculating the proportion of RBPMS cells that also contained GFP from three images (10× objective) captured arbitrarily across the flatmount retina (obtained on a Perkin Elmer Ultraview VOX imaging system using Volocity 6.3 software; *n*=5 animals per WT or NLS).

Inflammatory response in the retina was determined in cross-sectioned retinas immunolabelled with Iba1 and GFAP and imaged on a Perkin Elmer Ultraview VOX imaging system (Volocity 6.3 software; *n*=5 animals/genotype, four to five images from three sections spanning across each retina). A region of interest (ROI) across all retinal layers was quantitated for each image. Images were segmented using ImageSURF, a random forest classifier for ImageJ (O'Mara et al., 2017, 2019), which allows unbiased and reproducible image segmentation based on a classifier trained to recognise signal from background, and percentage area of labelling was quantitated using ImageJ (NIH v1.46) as a proxy for cell numbers (Collins et al., 2015; Fernandez-Martos et al., 2015).

Retinal layer thickness was determined from images of DAPI-stained retinal cross sections (Perkin Elmer Ultraview VOX imaging system using Volocity 6.3 software; *n*=5 animals/genotype, four images from three sections spanning across each retina) and quantitated in ImageJ to measure the thickness of total retina and each retinal layer: ganglion cell layer (GCL), inner plexiform later (IPL), inner nuclear layer (INL), outer plexiform layer (OPL), outer nuclear layer (ONL) and photoreceptor layer (PL) (Fig. 1B). Owing to potential differences in the sectioning plane between the retinas, thickness of each layer was divided by the total thickness of the retina (Dysli et al., 2015).

Synaptic inputs from bipolar and amacrine cells into RGCs were determined from images of the IPL layer of synaptophysin-immunolabelled retinal cross sections (Perkin Elmer Ultraview VOX imaging system using Volocity 6.3 software; *n*=5 animals/genotype, four images from three sections spanning across each retina). For analysis, a 3000 µm^2^ ROI was placed over the IPL in each image (dashed red line boxes in Fig. 4). A Gaussian blur (σ=2) was applied in ImageJ before image segmentation using ImageSURF (O'Mara et al., 2017, 2019). Images were subjected to watershed segmentation, and puncta from 0.15 µm^2^ to 2.0 µm^2^, as described previously (Mitew et al., 2013), were assessed to determine the average number/synaptic density, size and percentage area occupied by synaptophysin-positive puncta.

NFH localization in cell bodies of RGCs was quantitated in images of flatmount retinas immunolabelled with GFP (mouse antibody) and NFH (chicken antibody) (Perkin Elmer Ultraview VOX imaging system using Volocity 6.3 software; *n*=5 animals/genotype, five to six images spanning from central retina to peripheral retina). The number of NFH-positive cell bodies and the percentage of GFP cells with colocalizing NFH were manually counted and quantified by a researcher blinded to conditions.

Axon density in optic nerves was quantitated from images of semi-thin optic nerve sections (prepared for downstream electron microscopy analysis, *n*=4 per treatment) stained with Toluidine Blue (Zeiss Lab.A1 imaging system with Zen 2 software). Using ImageJ, a 1000 µm^2^ ROI was constructed over the central optic nerve, and the number of axons was determined using the AxonJ plugin (Zarei et al., 2016).

NFH-positive axons in the optic nerve were quantitated from images of immunolabelled optic nerve cross sections (Nikon Eclipse Ti2-N-WID imaging system with NIS Elements version 5.02 software; *n*=5 animals per treatment group; one image from three sections capturing the entire optic nerve). ROIs capturing the whole optic nerves were constructed. A Gaussian blur (σ=2) was applied in ImageJ before image segmentation using ImageSURF (O'Mara et al., 2017, 2019). Images were subjected to watershed segmentation, and puncta >0.15 µm^2^ were assessed to determine the percentage area occupied by NFH-positive axons and the average number of NFH-positive axons.

To ensure that the SC that was contralateral to the injected eye was assessed for Vglut-2 and Iba1 analysis, a subset of mice (*n*=5) underwent intraocular injection in the left eye with the fluorescently tagged anterograde tracer CTB. When injected into the eye, CTB is taken up by RGCs, through their axons and into terminals in the SC (Fig. 8A) (Angelucci et al., 1996). Coronal brain sections from these mice were immunolabelled with the presynaptic marker Vglut2 and the microglial marker Iba1 (*n*=5 animals per treatment; three to four brain sections from Bregma positions −3.80 mm to −4.48 mm). The visual layers of the SC were identified by Vglut2 labelling. For Vglut2 analysis, 3× 32.2mm^2^ fields of view were captured from the lateral to medial lateral portion of the SC (represented in Fig. 8A; Perkin Elmer Ultraview VOX imaging system using Volocity 6.3 software), and images were assessed (as per synaptophysin, above) to determine the average number, size and percentage area occupied by Vglut2-positive puncta. Iba1 immunolabelling within the entire SC was captured (Olympus Slide Scanner VS120 using Olympus VS-ASW-FL 2.8 software). An ROI was constructed over the side of the SC that was stained with CTB and applied to the corresponding Iba1-immunolabelled image. The same ROI was reversed and applied to the contralateral hemisphere so that Iba1 immunolabelling could be normalized. Images were segmented using imageSURF, and ImageJ Particle Analysis tool was used to quantify the percentage area occupied by Iba1-positive microglia.

TEM imaging was carried out on a Hitachi 7700 transmission electron microscope with a LaB6 filament, at 80 kV in high-contrast mode. From each optic nerve (*n*=4 animals per treatment), one good quality section in the proximal region and one in the distal region was selected. Within these sections, four photomicrographs (2000× magnification) across the section were obtained. Four×100 µm^2^ ROIs per photomicrograph (*n*=16×100 µm^2^ ROI across each optic nerve section, proximal and distal). The number of axons containing greater than three vesicles was manually counted. Data were separated into axons containing four, five, six, seven, eight and greater than eight vesicles. Histograms of these data were created.

#### Statistical analysis

Unless otherwise stated, statistical analysis was carried out using one-way ANOVAs with Tukey post hoc tests (GraphPad Prism 6). The difference between hTDP-WT-GFP and hTDP-ΔNLS-GFP in the percentage of GFP cells colocalizing with NFH expression in cell bodies in the retina was assessed with a two-tailed unpaired parametric Student's *t*-test. All data are presented as mean±s.e.m., with *P*<0.05 considered significant. For the optomotor data, linear mixed effects models were used for analysis. Q-Q and residual plots were used to determine normality of residuals and homogeneity of variance to satisfy the assumptions of a linear mixed effects model. Random intercepts and slopes were fitted for each animal to account for variance not attributable to the effect of treatment, enabling the assumption of independence to be relaxed for this repeated measures experiment. The R environment (R Foundation for Statistical Computing) and lme4 package (Bates et al., 2015) were used to fit the model using restricted maximum likelihood estimation.

## Supplementary Material

Supplementary information
