# Supplementary material for: TDP-43 mislocalization drives neurofilament changes in a novel model of TDP-43 proteinopathy
Source: Dis Model Mech. 2021 Feb 11;14(2):dmm047548. doi: 10.1242/dmm.047548 (PMC7888715; doi:10.1242/dmm.047548)
Supplement: Supplementary information [file dmm-14-047548-s1.pdf]

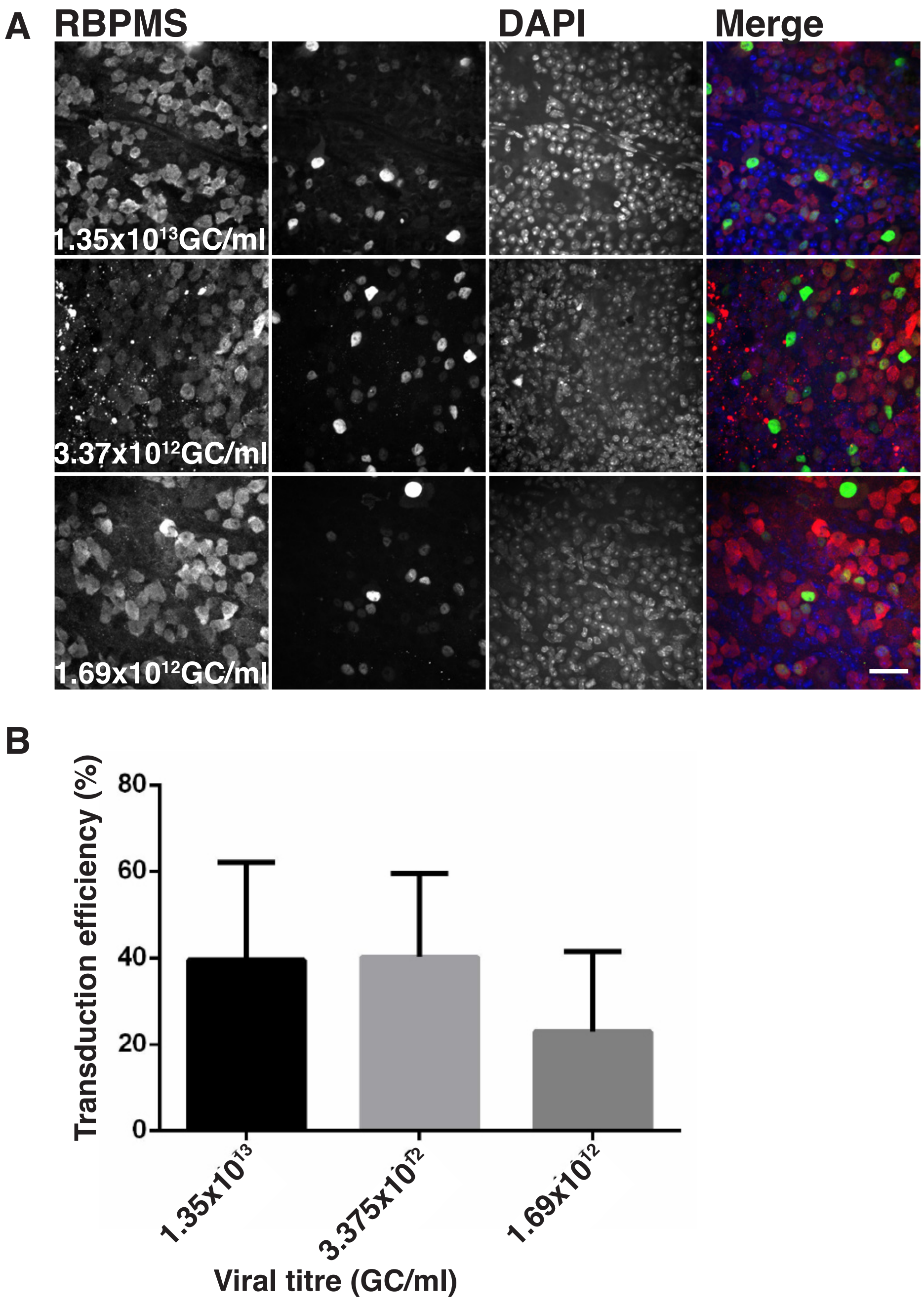

Fig. S1. Optimization of RGC transduction with TDP-WT-GFP. (A) Wholemount retina from mice injected with hTDP-WT-GFP at different concentrations, immunolabelled for RBPMS (red) GFP (green), and stained with DAPI (blue). (B) The percentage of co-localising GFP and RBPMS RGC bodies were quantified to provide an estimate of viral transduction efficiency for each concentration. Results are mean and SEM. Scale bar 20μm.

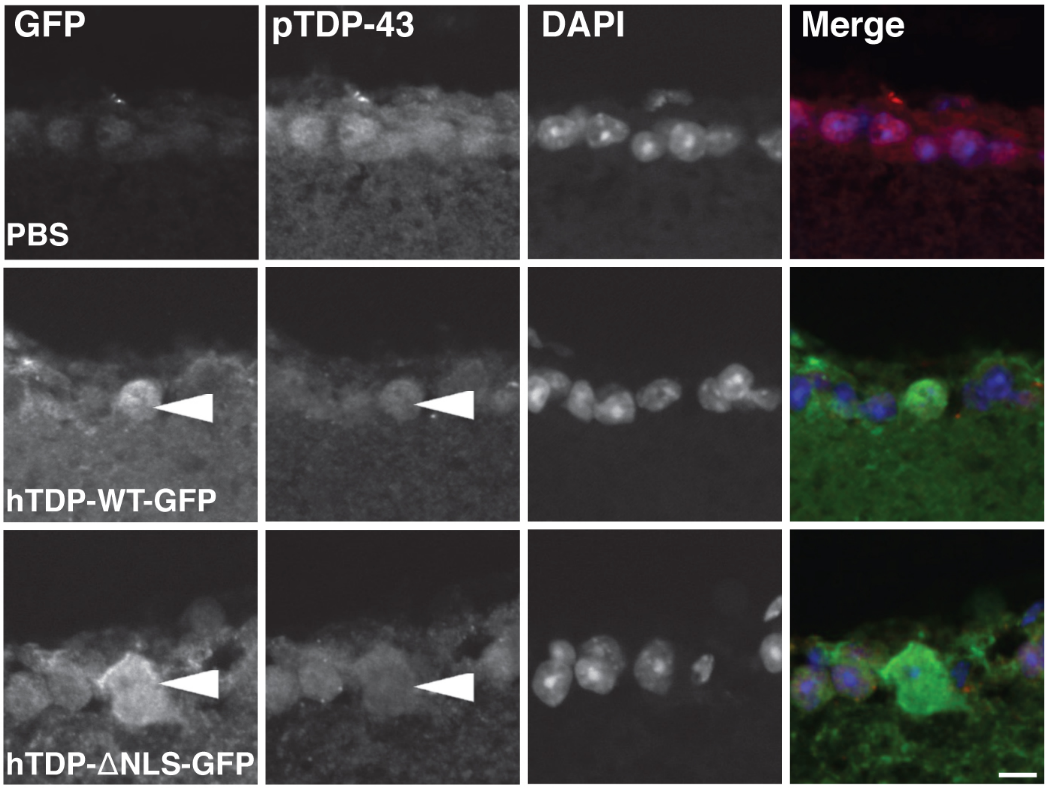

Fig. S2. Phosphorylated TDP-43 in cross-sectioned retina. Cross-sectioned retinas from mice injected with PBS vehicle, hTDP-WT-GFP, hTDP-ΔNLS-GFP immunolabelled for phosphorylated TDP-43 (at residues serine 409/410, red), GFP (green) and stained with DAPI (blue), showing minimal phosphorylated TDP-43 labelling GFP-positive cells (arrowheads) or in other cells in the RGC layer. Scale bar 20μm.

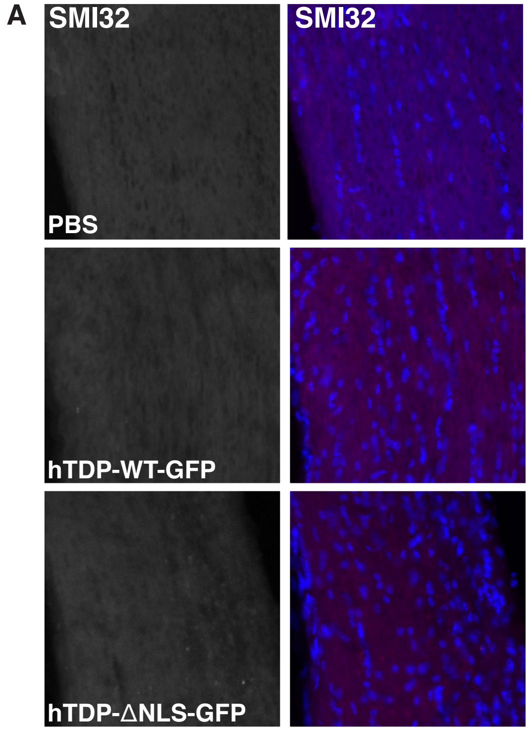

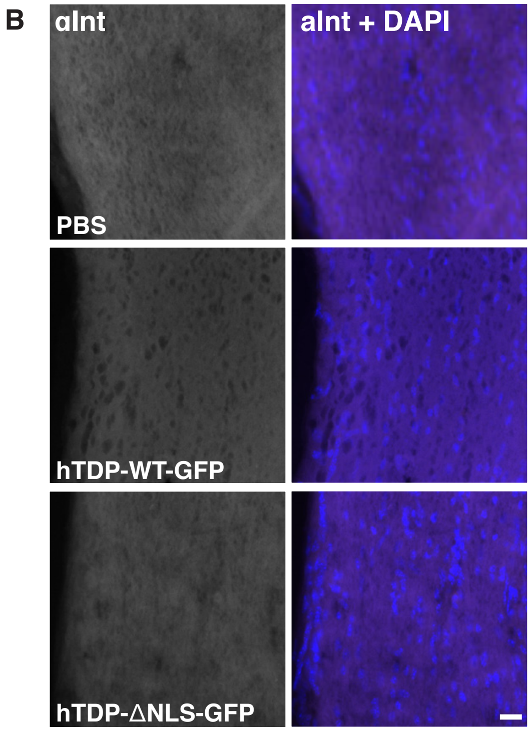

Fig. S3. SMI32 and alpha internexin immunolabelling in longitudinal optic nerve sections. Longitudinally sectioned optic nerves from mice injected with PBS vehicle, hTDP-WT-GFP, hTDP-ΔNLS-GFP. (A) Tissue immunolabelled for SMI32 (red) and stained with DAPI (blue), showing absence of SMI32-positive labelling, and similarly distributed NFH-labelling. (B) Tissue immunolabelled for alpha internexin (red) and stained with DAPI (blue), showing absence of alpha internexin-positive labelling. Scale bar 20μm.

Disease Models & Mechanisms • Supplementary information
